# Use of Polycaprolactone Electrospun Nanofibers as a Coating for Poly(methyl methacrylate) Bone Cement

**DOI:** 10.3390/nano7070175

**Published:** 2017-07-10

**Authors:** Morshed Khandaker, Shahram Riahinezhad, Harsha G. Jamadagni, Tracy L. Morris, Alexis V. Coles, Melville B. Vaughan

**Affiliations:** 1 Center for Interdisciplinary Biomedical Education and Research, University of Central Oklahoma, Edmond, OK 73034, USA; MVaughan4@uco.edu; 2 Department of Engineering & Physics, University of Central Oklahoma, Edmond, OK 73034, USA; sh_riahi@yahoo.com (S.R.); hjamadagni@uco.edu (H.G.J.); 3 Department of Mathematics and Statistics, University of Central Oklahoma, Edmond, OK 73034, USA; TMorris2@uco.edu; 4 Department of Biology, University of Central Oklahoma, Edmond, OK 73034, USA; acoles@uco.edu

**Keywords:** poly(methyl methacrylate), polycaprolactone, bone cement, electrospun, nanofiber

## Abstract

Poly(methyl methacrylate) (PMMA) bone cement has limited biocompatibility. Polycaprolactone (PCL) electrospun nanofiber (ENF) has many applications in the biomedical field due to its excellent biocompatibility and degradability. The effect of coating PCL ENF on the surface topography, biocompatibility, and mechanical strength of PMMA bone cement is not currently known. This study is based on the hypothesis that the PCL ENF coating on PMMA will increase PMMA roughness leading to increased biocompatibility without influencing its mechanical properties. This study prepared PMMA samples without and with the PCL ENF coating, which were named the control and ENF coated samples. This study determined the effects on the surface topography and cytocompatibility (osteoblast cell adhesion, proliferation, mineralization, and protein adsorption) properties of each group of PMMA samples. This study also determined the bending properties (strength, modulus, and maximum deflection at fracture) of each group of PMMA samples from an American Society of Testing Metal (ASTM) standard three-point bend test. This study found that the ENF coating on PMMA significantly improved the surface roughness and cytocompatibility properties of PMMA (*p* < 0.05). This study also found that the bending properties of ENF-coated PMMA samples were not significantly different when compared to those values of the control PMMA samples (*p* > 0.05). Therefore, the PCL ENF coating technique should be further investigated for its potential in clinical applications.

## 1. Introduction

Cemented fixation of an implant, used for both osteoporotic and osteoarthritic bone diseases, requires bone cement to hold the implant in place. Although considerable advances have already been made to improve the biological performance of cement, the ideal long-term mechanical stability of a cemented implant has still not been achieved. An ideal cementing material for cemented surgeries should have surface energy and mechanical interlock to ensure a long-lasting fixation between the implant-cement and the cement-bone interfaces [1,2,3,4]. The critical task for creating a long lasting tissue-implant interface resides in achieving the functional integration to mimic the native tissue-tissue failure response [5]. Appropriate mechanical interlock and adequate osseointegration is present between the joining tissues at the natural tissue-tissue interfaces. Since bone cement is a bio-inert material, in the case of the natural tissue-cement interface in a cemented joint, the joining of cement with bone is achieved by mechanical interlock. The goal of this research is to increase the osseointegration at the tissue-cement interface by improving the bioactivity of cement so that it will mimic the native tissue-tissue failure response under functional loading.

Nanofibers are a simple, scalable, inexpensive, and supplementary surface treatment method for biomaterials that have been implemented by various researchers [6,7,8]. Most research on nanofiber applications with cement is focused on improving the mechanical properties of cement rather than on improving the bioactivity of bone cement. For example, Wagner and Cohn [7] used high performance polyethylene fibers as a reinforcing phase in Poly(methyl methacrylate) (PMMA) bone cement. The authors found that the surface coating treatments of the Spectra 900 polyethylene fibers apparently did not significantly affect the mechanical properties of the PMMA bone cement. Saha and Pal [6] found that the addition of 1–2% by weight of graphite and up to 6% of aramid fibers into PMMA cement significantly reinforced the mechanical strength of PMMA. However, the previous authors did not conduct cell viability studies to evaluate the effect of their fiber treatments on the biocompatibility of PMMA. Nanofibers can be biomineralised by the immobilization of functional proteins and minerals with the fiber. Wu et al. [9] produced aligned poly(l-lactide)/poly(methyl methacrylate) binary blend fibers and mats loaded with a chimeric green fluorescence protein having a bioactive peptide with hydroxyapatite binding and mineralization properties by pressurized gyration. The previous authors’ research showed that nanofibers can have controllable inherent mineralization abilities through integrated bioactivity. The increase of the bioactivity of a bone cement using an electrospun nanofiber coating on cement has not yet been investigated and is pursued in this study.

Electrospinning is a process by which fibers with micron- to nanometer-sized diameters can be deposited on a substrate from an electrostatically driven jet of polymer solution through a needle [10,11]. These fibers have a high surface area-to-volume ratio, which can be used to produce an electrospun nanofiber (ENF) membrane for biomedical applications [11,12]. Polycaprolactone (PCL) nanofibers can be produced using an electrospinning process that is biocompatible and nontoxic [11,13]. We have recently developed a PCL ENF that can control the flow of cement into bone cavities using our patented electrospun nanofiber technology [14]. The effect of the PCL ENF coating on PMMA is not known. A thorough understanding of the PCL ENF coating structure (e.g., topography, thickness) and biomechanical function (cytocompatibility and mechanical responses) relationship in vitro is necessary to evaluate the efficacy of the PCL ENF coating as a functional bio-coating for cemented implant surgery.

The hypothesis of the study is that the PCL ENF coating on cement may increase the biocompatibility of the cement and may lead to better mechanical stability of the cement with the adjoining bone tissue. To test this hypothesis, the first objective of this study was to evaluate the in vitro effects of the PCL ENF coating on the surface topography and cytocompatibility (osteoblast cell adhesion, proliferation, mineralization, and protein adsorption) properties of PMMA bone cement. The second objective of this study was to determine the effect of the PCL ENF coating on the mechanical properties of the PMMA bone cement under three-point bend loading.

## 2. Results

### 2.1. Surface Topography

**Figure 1:**
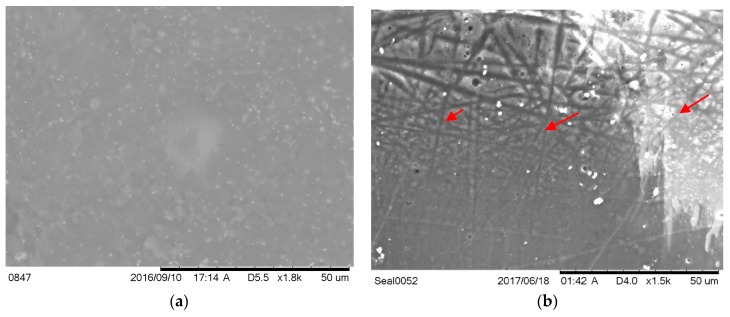
Scanning electron microscope images of the (**a**) control and (**b**) electrospun nanofiber (ENF) coated Poly(methyl methacrylate) (PMMA) specimen. The red arrows in Figure 1b show the embedded single Polycaprolactone (PCL) ENF in the ENF coated PMMA specimen.

**Figure 2:**
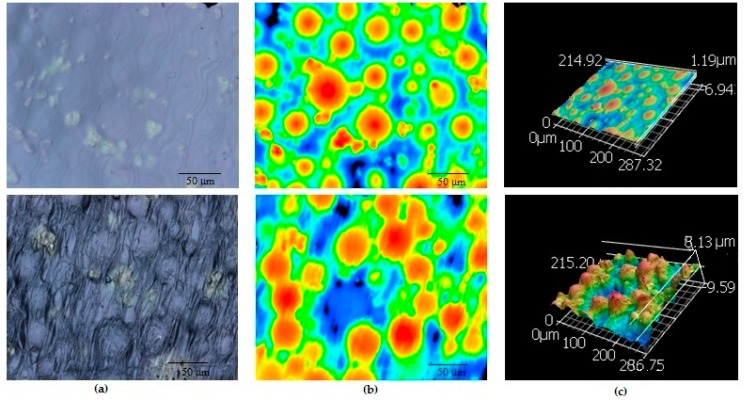
Confocal microscope images of a control (top row), and ENF coated PMMA (bottom row) sample: (**a**) 2D topography; (**b**) height amplitude mapping; and (**c**) 3D topography images. The length of the scale bar is 50 micrometers in the figures of column (**a**,**b**). The magnification of column (**c**) images is 20×.

A clearly visible difference in surface topography was observed from SEM (Figure 1) and confocal microscope images (Figure 2) between the control and ENF coated PMMA samples. Significant differences in mean roughness parameters were observed between the sample groups (*p*-value < 0.05) (Table 1). This result suggested a significant change of the surface texture due to the ENF treatment. The overlapping of multiple fibers was observed in the ENF coated PMMA samples (Figure 1b). The fiber thickness calculated from the SEM images of the ENF coated samples was found to be in the range from 0.432 to 0.648 μm. The 2D and 3D confocal topography images of the ENF coated PMMA samples showed that the direction of ENF deposition was mainly uni-directional, although it was not possible to measure the quality of the fiber alignment for the images due to embedding of the fiber into the PMMA cement. It is clear from comparing the confocal images between the control and ENF coated PMMA samples that the visibility of PMMA beads increased due to the deposition of ENF on the PMMA cement.

### 2.2. Cytocompatibility Properties

**Figure 3:**
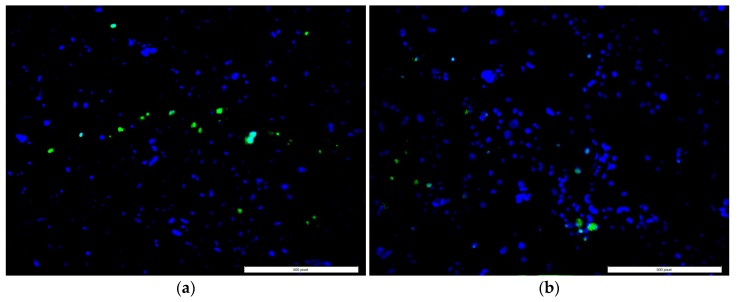
A representative fluorescent stained image (100× total magnification, scale bar = 500 μm) from adhesion and proliferation assays showing adhered (blue color) and proliferated cells (green color) of the (**a**) control and (**b**) ENF coated PMMA samples.

**Figure 4:**
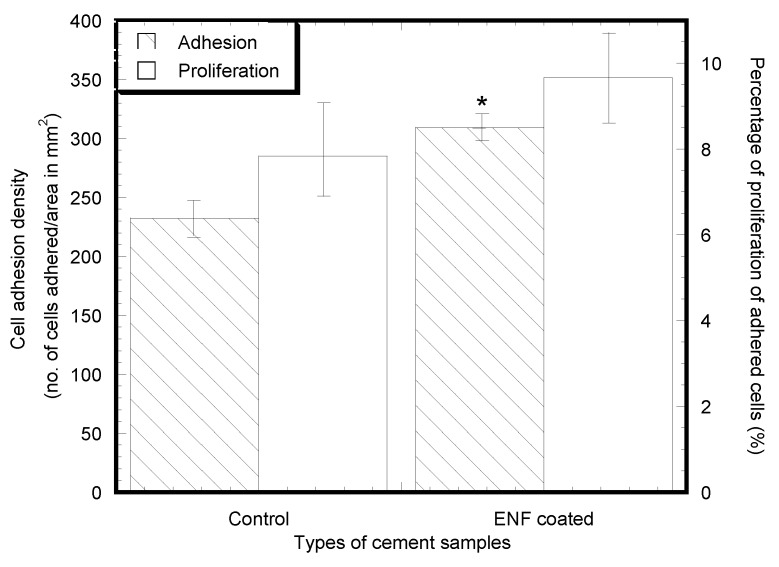
Mean cell adhesion density (±standard error) and the percentage of cell proliferation (±standard error) for the control and ENF coated PMMA groups after 48 h of cell culture. Data are presented with *n* = 14 for both samples. Note: * *p* < 0.05 (compared to control).

Cell adhesion and proliferation successfully occurred on the surface of the control and ENF coated PMMA samples, as shown in Figure 3. There was a significant difference in the mean cell densities (combined blue and green nuclei) of the control and ENF treated PMMA sample groups observed with time (*F*_1,56_ = 16.18, *p*-value < 0.001) (Table 2). Both samples demonstrated adhered cell nuclei (blue color Hoechst stained) and proliferating cell nuclei (green color edu-click stained). Cells grew more readily on fibrous samples in ENF coated PMMA samples and with increased cluster distribution along the surface of the fibers on the ENF coated PMMA surface compared to the control (*p*-value < 0.001). Although the mean number of cells proliferating on the PMMA surface was higher for the ENF coated PMMA samples compared to the control samples, there was no significant difference in the mean percentage of cell proliferation between the samples (*p*-value = 0.297) as shown in Figure 4. These results suggest that the ENF coating may have a positive influence on the in vitro osseointegration with the PMMA surface.

**Figure 5:**
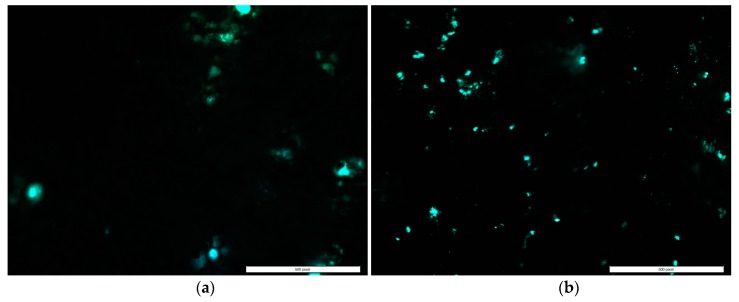
A representative fluorescent stained image (100× total magnification, scale bar = 500 μm) from the mineralization assays showing the released mineral (green) from the cells of the (**a**) control and (**b**) ENF coated PMMA samples after 3 weeks of cell culture.

**Figure 6:**
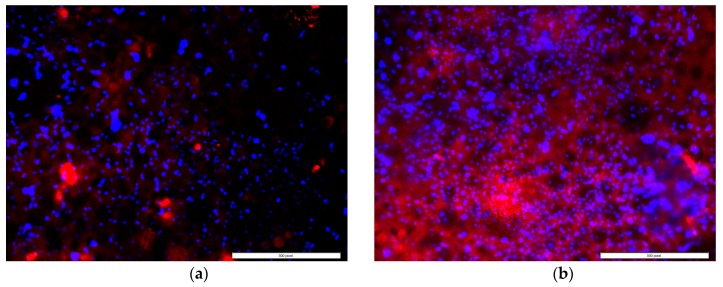
A representative fluorescent stained image (100× total magnification, scale bar = 500 μm) from the protein adsorption assays showing osteoblast nuclei (blue) and released osteonectin (red) from the cells of the (**a**) control and (**b**) ENF coated PMMA samples after 3 weeks of cell culture.

**Figure 7:**
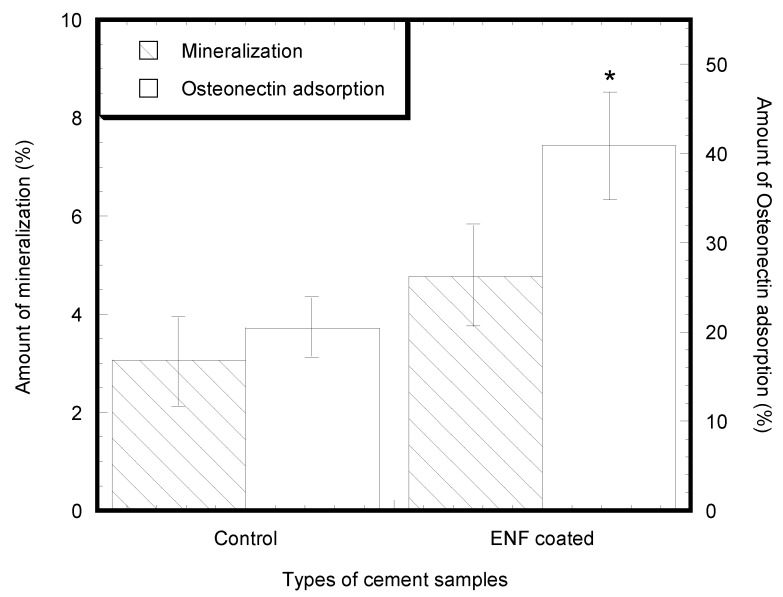
Mean amount of mineralization (±standard error) and mean amount of osteonectin (±standard error) for the control and ENF coated PMMA groups. Note: * *p* < 0.05 (compared to control).

Cell mineralization and protein adsorption successfully occurred on the surface of all types of PMMA samples as shown in Figure 5 and Figure 6. Both samples demonstrated adhered cells (blue nuclei) and hydroxyapatite mineralization from the cells (green stained). Although the study found a low amount of mineralization for both the control (0.18–11.32%) and ENF coated PMMA (0.39–10.79%) samples, the mean number of cells mineralized after adherence to the PMMA surface was higher for the ENF coated PMMA samples compared to the control samples, and there was no significant difference in the mean amount of mineralization (total green stained area × 100%/area of image field) of the control and ENF treated PMMA sample groups (*p*-value = 0.228) (Figure 7). Both samples demonstrated adhered cells (blue nuclei) and the presence of osteonectin from the cells (red stained). There was a significant difference in the mean amount of osteonectin (total red stained area × 100%/area of image field) between the control and ENF treated PMMA sample groups (*p*-value = 0.008) (Figure 7). The result suggested that the inclusion of ENF has a positive effect on the cytocompatibility properties of PMMA.

### 2.3. Mechanical Tests

**Figure 8:**
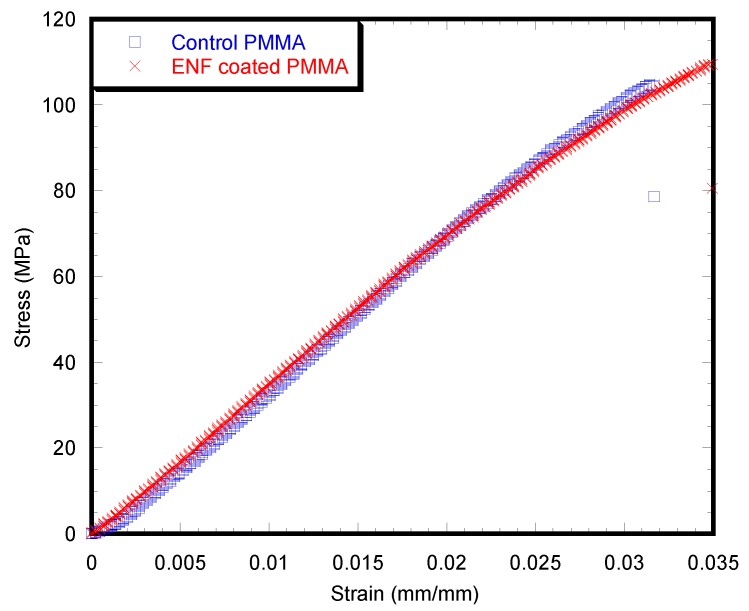
Typical stress vs. strain plots of the control and ENF coated PMMA specimens. The observed difference between the maximum stress and strain values in the figure is due to the difference of the dimensions and internal structures of the samples.

There was negligible difference of the stress-strain behavior between the control and ENF coated samples (Figure 8). The stress-strain curves for both samples can be characterized as an initially elastic response, followed by a short inelastic region and then a sudden descending response due to failure of the specimen. Table 3 summarizes the bending strength, *σ*_f_, bending modulus, *E*, and maximum deflection of the control and ENF coated PMMA samples. Although the mean value for each of the experimental parameters under bending load was different between the ENF coated and control PMMA samples, the difference was not statistically significant (*p*-value > 0.05).

## 3. Discussion

The topographical analysis results (Figure 1 and Figure 2 and Table 1) suggested that the inclusion of ENF has a significant effect on the surface morphology of PMMA. There was variability in the amount of fiber deposition, alignment, and diameter of the fiber along the ENF coated PMMA surface. This variability happened due to the variation of the fiber production and manual collection process. The topographical difference among the ENF coated PMMA samples led to a higher amount of variance (represented by the standard error) in roughness parameters for the ENF coated PMMA samples compared to the control samples (Table 1). To reduce the effect of the fiber topography on the cytocompatibility and mechanical test results for the ENF coated PMMA samples, fibers were deposited on the parallel wire collectors for the same time period under the same fiber production conditions (PCL solution viscosity, DC voltage, and solution flow rate). An automated fiber collection process from the parallel wire collectors is required to further minimize the effect of topographic variation on the cytocompatibility and mechanical test results of the ENF coated PMMA samples.

The ENF coated PMMA samples demonstrated better biological responses compared to non-treated PMMA samples. Over the three-week period of in vitro culture, osteoblasts persisted, proliferated, and differentiated on the PMMA substrate in the presence and absence of ENF. The adhesion and proliferation test results of control PMMA samples were similar to our previously published report [15]. The mean percentage of proliferation in the control versus ENF coated PMMA was not significantly different. This suggests that the rate of proliferation was constant in both treatments. However, more cells were clearly present in clusters for the ENF coated PMMA compared to the control (Figure 3). Two hypotheses can be provided for this response: (1) When cells were initially plated, they adhered better to the ENF matrix and therefore more cells were present initially; or (2) Cells were dying at a greater rate in PMMA compared to the ENF coated PMMA. Neither of these hypotheses were tested here, but would be interesting to study in the future.

Osteoblast cell differentiation was demonstrated by calcium phosphate mineral (Figure 5) and osteonectin expression from fluorescent stained images (Figure 6). While both mineralization and osteonectin expression increased with the ENF treatment, the increase of mineralization was not found to be statistically significant for the ENF coated PMMA group compared to the control (Figure 7). The study found a low percentage amount of mineralization for both the control and ENF coated PMMA samples. This is due to the fact that the study conducted the mineralization assay after 14 days of cell culture on the samples according to the vendor recommendation (OsteoImage™ mineralization assay kit from Lonza). Since the amount of mineralization from the adhered cells are highly dependent on the period of cell culture [16,17], more culture time is therefore required to obtain a higher amount of mineralization.

In this study, we observed increased cytocompatibility properties (adhesion, proliferation, and protein adsorption) of the ENF coated PMMA implants compared to PMMA. This is because higher cell functions were created via better cell signaling arising from the cell-cell contact and the cell-ENF components in the ENF coated PMMA samples. Cell signals depend upon the physical (micro- or nano-structured surface topography, composition of ENF) and chemical properties of ENF. There exists differences of the physio-chemical properties between the control and ENF coated PMMA samples. The PCL nanofibers on PMMA lead to different physical characteristics viz. porosity and density due to the distribution of the PCL fiber. PCL in the ENF coated PMMA created a larger surface area that provided more cell binding sites. Additionally, PCL ENF can absorb numerous proteins or minerals akin to a cell membrane receptor, thus favoring cytocompatibility properties for the ENF coated PMMA samples compared to the control.

PMMA is a bio-inert material. The purpose of coating PMMA at the bone/cement interface was to improve the biocompatibility of the PMMA cement without diminishing the mechanical properties of PMMA in in vivo conditions. The stress-strain behavior (Figure 8) and the measured mechanical properties (Table 3) of the PMMA and ENF coated PMMA samples suggested that the inclusion of ENF has no statistically significant effect on the mechanical property of PMMA under three-point bend loading. Therefore, the PCL ENF membrane can potentially be used as a functional bio-coating material for PMMA. Since the PCL ENF membrane has negligible strength (*E* = 10.2–27.3 MPa and σ_f_ = 1.5–3.6 MPa [18]) compared to PMMA, it has a negligible impact on the increase of the strength of PMMA. An alternative high strength electrospun nanofiber material such as Polycaprolactam (nylon 6), or graphene oxide-based nanofibers can be used with PMMA for the combined improvement of mechanical and biological properties. The mechanical properties of PCL ENF depended highly upon the number of electrospun layers. This study used only 24 layers of fiber (negligible weight compared to the weight of the PMMA block) to coat the PMMA for the mechanical test samples. A large amount of PCL ENF is required to improve the elasticity of PMMA.

This study was motivated by a clinical problem where the heterogeneous flow of bone cement around the implants to the adjacent bone tissue has been observed due to the porosity of bone [14]. Since significantly more cracks are associated with the interdigitated area and the cement/bone interface than with the implant/cement interface [19], there is a high probability that localized fractures may occur at the narrowly confined cement/bone interfaces [20] due to this heterogeneous flow of cement. The goal of reducing the localized fractures due to the heterogeneous flow of bone cement at the tissue-cement interface by a functional nanofiber coating on cement has been investigated in a separate study.

The method of coating the bone cement by the ENF membrane can be applied in clinical fields for the fixation of implants in bone using PMMA. For a case of cemented implant fixation, an electrospinning process (patent pending) has been used in our study to create a cylindrical PCL ENF cup by electrospinning PCL on a round shape collector [14]. In our in vivo study, we have inserted the PCL ENF cylindrical cup into the hole of a rabbit femur at the epiphyso-metaphyseal junction. The cement in the dough phase of mechanical properties during the polymerisation process was injected into the hole of the ENF cup by a syringe. Subsequently, the implant was hand-pressed into the cement. Due to the high flexibility and porosity of the ENF cup, the cement with ENF anchored with the bone. Our patent pending method [19] can also be applied to a cemented hip implant, where the shape and size of the ENF membrane can be the same as the shape and size of the hole drilled for the anchor of the cemented hip implant. Our invented ENF cup succeeded in holding the cement, which was confirmed from mechanical and histological tests. The results of the mechanical and histological tests will be presented in a separate manuscript.

To our knowledge, this is the first study to evaluate the effect of an aligned PCL ENF coating treatment on the cytocompatibility of PMMA using osteoblast cells and flexural properties of PMMA cement. However, the value of the *R_a_* of PMMA (0.26 ± 0.03 μm) in this study is in close agreement with the *R_a_* of PMMA (0.37 ± 0.09 μm) found by Moursi et al. [20]. The values of the mechanical and cytocompatibility properties of PMMA in this study are different than those values of PMMA in our previous study [15], likely because we used two different brands of PMMA cement. Cobalt ^HV^ bone cement was used in our previous study, whereas Stryker Simplex^®^ P bone cement was used in this study. Due to the ease of sample preparation, this study was limited to only bending tests on the test samples to measure the effect of the PCL nanofibers on PMMA. Another reason for using flexural testing is that it is more sensitive to surface effects than tension and compression.

This study is limited to the production of PCL nanofibers by an electrospinning method and blending of the produced PCL ENF with PMMA bone cement. There are several other methods, such as pressurized gyratory spinning [21] and pull spinning [22], which can be used to produce a PCL nanofiber membrane for the coating of bone cement. The capability of electrospinning to function as an automated, scaled, and point-of-use fiber manufacturing platform was demonstrated by Khandaker and Shahram [23]. There might be positive and negative consequences from using each of the above methods for the rapid coating of bone cement with nanofibers, which would be a potential area of investigation related to this research. This study is limited to the use of PCL nanofibers as the coating material. The reason for selecting PCL ENF among many other biocompatible and degradable nanofibers is that PCL can be immobilized with osteoconductive biomolecules, antimicrobial nanoparticles, growth factors, and proteins. The blending of protein and mineral immobilized fibers with PMMA cement can further improve the bio-functional properties of PMMA cement. Another limitation of the study was that the mechanical tests were conducted on samples kept at room temperature. However, bone cement undergoes polymerization due to the inclusion of a system that generates free radicals, but once in the body further polymerization occurs due to the body temperature, thus the cement should have been left in saline or cell culture fluid for a week at 37 °C before mechanical testing to allow the cement to be set as it would be in the body. Considering the fact that there is no effect of the PCL ENF coating on PMMA, and that PCL fiber is degradable, it is anticipated that keeping the samples at 37 °C in a phosphate buffer solution would have no effect on the mechanical properties. Further research is required to verify this anticipation.

The cytocompatibility properties of nanoparticle (MgO, hydroxyapatite, chitosan, BaSO_4_, SiO_2_) incorporated PMMA was explored in our earlier study [15]. Several researchers were able to attach drugs (e.g., collagen [24], ampicillin [25], resveratrol [8], hydroxyapatite [26]) with nanofibers. The combined application of nanoparticles and nanofibers blended on PMMA can further enhance the biocompatibility of PMMA, which is a potential area of new research.

## 4. Materials and Methods

### 4.1. Materials

A Surgical Simplex^®^ P radiopaque bone cement without antibiotic package was used as the PMMA cement. The bone cement package contains 40 g of PMMA powder consisting of 6 g of polymethyl methacrylate, 30 g of methyl metacrylate-styrene-copolymer (contains benzoyl peroxide and barium sulfate), and 20 mL of liquid that consists of 19.5 mL of methyl methacrylate, *N*,*N*-dimethyl-para-toluidine, and hydroquinone. PCL pellets (pellet size ~3 mm, average M_n_ 80,000) and acetone (laboratory reagent ≥ 99.5%) were purchased from Sigma Aldrich (Sigma-Aldrich Co., LLC., St. Louis, MO, USA).

### 4.2. Sample Preparation

#### 4.2.1. Sample Design

Two groups of samples were prepared for surface topography, mechanical, and cytocompatibility tests to achieve the objectives of this study. The groups are PMMA only (control) and aligned PCL electrospun nanofiber (ENF) coated PMMA cement, referred to in this article as ENF coated PMMA. The total number of samples tested for confocal, cytocompatibility, and mechanical tests per group are listed in Table 1, Table 2 and Table 3, respectively. The SEM images were captured for only one sample per group.

#### 4.2.2. Sample Fabrication Process

**Figure 9:**
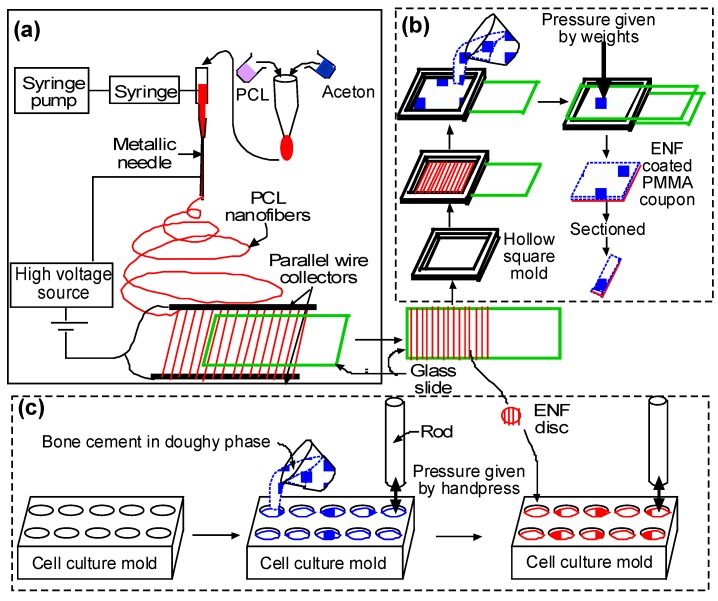
Schematic representation of the production methods for (**a**) single layer aligned PCL ENF production; (**b**) surface characterization/mechanical test samples; and (**c**) cytocompatibility test samples.

The process of fabrication for the confocal, cytocompatibility, and mechanical test samples are depicted in Figure 9. Each step in the fabrication process is explained in the following paragraphs.

##### (a) Fiber Production

**Figure 10:**
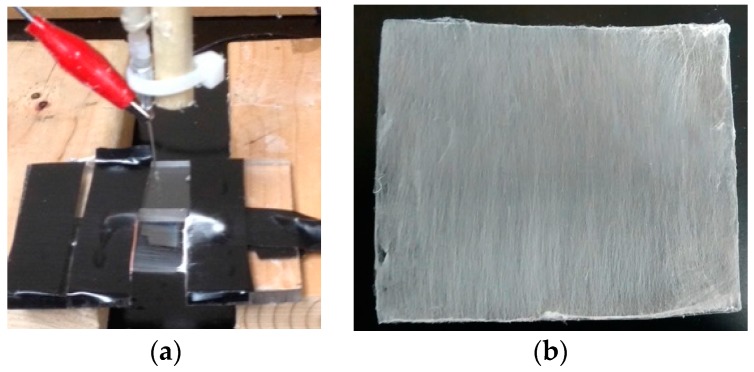
(**a**) Fabrication of aligned PCL fibers for coating using the electrospinning process; (**b**) Collected fibers on a glass slide.

A glass slide (25 × 75 × 1 mm) was coated with aligned PCL nanofibers using an electrospinning setup. The details of the PCL electrospun nanofiber fabrication can be found in Khandaker and Shahram [24]. In short, PCL pellets (7.69 wt %) were mixed with acetone in an ultrasonic mixer (Sonics & Materials, Inc., Newtown, CT, USA). The sonication process was carried out at approximately 60 °C for 30 min. The solution was poured into a glass syringe on an infusion pump (Harvard Apparatus, mode # PHD ULTRA) for the PCL fiber production. The PCL solution was ejected from the glass syringe through an electrically-charged needle (23 G blunt needle, 25 mm length, model # BX 25). The needle was positively-charged by a high voltage (15 kV) DC power source (Gamma High Voltage Research, Inc., model # ES 30 series) and two parallel wires were negatively-charged. The aligned PCL fibers were collected between the two parallel wires (Figure 10a). To collect multiple layers of aligned fiber, the top surface of the glass slides touched the aligned fiber stream, moved up, and then moved forward to repeat the process to collect 24 layers of fibers (~1.6 micrograms) on the glass sides (Figure 10b).

##### (b) Surface Characterization and Mechanical Test Samples

**Figure 11:**
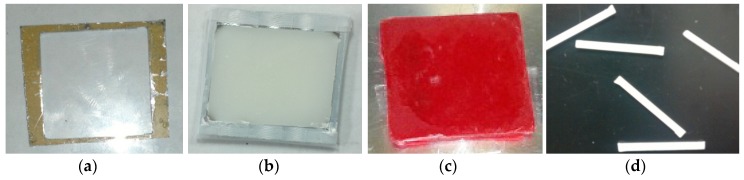
(**a**) Fabricated mold used for curing cement; (**b**) A cured PMMA cement in the mold; (**c**) A red dyed ENF coated cement sample on the chamber of the Hitachi TM 1000 scanning electron microscope; and (**d**) ASTM standard three-point bend PMMA cement specimen which was sectioned from the cured cement block.

A glass slide without and with PCL ENF was secured on the bottom of the mold using double-sided tape to prepare the control and ENF coated cement samples, respectively. According to the manufacturer recommendations, the PMMA solution was prepared by hand mixing 2.2 g of PMMA powder with 1.1 mL of methyl methacrylate (MMA) monomer using a powder:monomer ratio of 2:1. All solutions were cured in a custom-made aluminum mold (Figure 11a) to prepare a solid block of PMMA sample of size 25 × 20 × 2 mm. Cement was poured into the chamber of the mold. Another glass side was placed on top of the mold. Weights were stacked on the mold to cure the cement under 60 kPa pressure (clinically applied pressure during orthopedic surgeries [27]). The pressure was initiated at exactly three minutes after the onset of mixing and was sustained throughout the curing period (approximately 15 min) [28]. Figure 11b shows a cured control PMMA sample in a mold.

This study prepared three blocks of control and ENF coated PMMA samples for the mechanical tests. The 20 × 25 × 2 mm control PMMA blocks were used for both scanning electron microscopy (SEM) imaging and mechanical tests. Mechanical test blocks were also used for the surface topographical analysis using confocal microscopy. Since both PCL ENF and PMMA cement have a white color, this study prepared separate 20 × 25 × 2 mm ENF coated PMMA blocks for SEM imaging and mechanical tests. To prepare an ENF coated PMMA sample for SEM imaging, the PMMA solution was mixed with a red-colored dye before being poured into the mold. Figure 11c shows ENF coated PMMA samples that were used for SEM analysis. To prepare ASTM F417-78 standard flexural [29] test samples, (20 × 4 × 2) mm blocks were cut from the (20 × 25 × 2) mm block using a Buehler Isomet low-speed cutter. A (102 × 0.31 × 12.7) mm wafering blade was used for cutting the samples. Figure 11d shows the PMMA samples that were used for the mechanical tests. The samples were stored in cell culture flasks at room temperature for SEM analysis and mechanical tests.

##### (c) Cytocompatibility Test Samples

**Figure 12:**
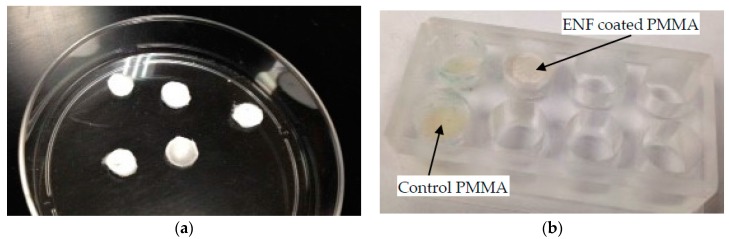
(**a**) ENF discs which were pressed on the top of PMMA during the doughy phase of the cement; (**b**) Control and ENF coated PMMA samples in an acrylic mold which were used for cytocompatibility tests.

Cytocompatibility properties (osteoblast cell adhesion, proliferation, mineralization, and protein adsorption) of the control and ENF coated PMMA samples were conducted in a custom made well. PCL ENF were collected between the wires until a fibrous cloth appeared. A 10 mm diameter PCL fiber disc was cut from the cloth using a punch (Figure 12a). PMMA specimens were prepared by mixing 0.5 g of PMMA beads with 0.25 mL of MMA. All PMMA samples, while still pliable, were divided into 4 parts by a knife and were poured in the well. Each part of the samples was hand pressed during curing by a flat-ended 9.565 mm diameter highly polished round bar. The round bar has clearance fits on the wells of the well plate. To prepare the ENF coated PMMA sample, a 10 mm diameter PCL fiber disc was placed on the cement and again pressed by the round bar to attach the PCL fiber on the top of the PMMA. The sample wells were kept sterile in a biological safety cabinet under ultraviolet (UV) light for subsequent cell culture.

### 4.3. Experiments and Analysis

#### 4.3.1. Surface Topography

Surface topography is an important parameter that plays a significant role in implant-bone adhesion. The influence of the ENF treatment on the surface morphology of PMMA was evaluated by a Keyence VK laser confocal microscope using 50× brightfield conditions. Scanning was conducted over 287.32 μm length × 214.93 μm width × 11.14 μm height for all samples. Topography images were compared for three control and ENF coated PMMA samples. Roughness parameters (*R_a_*, *R_z_*, and *R_sum_*) were directly measured from the line and surface scanning for the captured images [30].

#### 4.3.2. Cell Adhesion, Proliferation, Mineralization, and Protein Adsorption Tests on PMMA Samples

Rat osteoblast cells (R-OST-583; Lonza) were cultured at log phase growth in standard culture conditions (37 °C in a 5% CO_2_ incubator on tissue culture dishes) using DMEM/high glucose + 5% FBS and 1% ABAM (Sigma Chemical). Cells were dissociated using 1× trypsin/EDTA solution (Sigma Chemical) for 5 min at room temperature, followed by serum inactivation. Cells were counted using a hemocytometer, collected by centrifugation, and re-suspended at a concentration of 25,000 cells per 400 microliters of growth media as the custom-made acrylic well capacity was 400 microliters. Osteoblast cells were seeded at a density of 25,000 cells/well on each group of PMMA samples in a custom-made silicone well-plate. Cells were then cultured for 48 h to allow cell adhesion and proliferation on the PMMA surface. Parallel samples similar to those tested for adhesion and proliferation were cultured for 3 weeks and prepared for immunostaining to determine hydroxyapatite mineralization and osteonectin adsorption. A Click-iT^®^ EdU stain was used to evaluate cell adhesion and proliferation for each sample according to the vendor’s protocol [31]. This 48-h assay involved the addition of EdU, or 5-ethynyl-2′-deoxyuridine, to each well after the initial 24 h incubation. The EdU was a modified thymine nucleotide that contained a terminal alkyne. After a total of 48 h, the cells were fixed with paraformaldehyde and stained with Alexa-488. The terminal alkyne in the EdU reacted with the azide in Alexa-488, which caused the proliferated cells that incorporated the EdU tag to fluoresce green under fluorescent microscopy. An OsteoImage™ mineralization assay kit from Lonza was used according to the vendor’s protocol. For the protein adsorption test, anti-osteonectin (clone AON-1; Developmental Studies Hybridoma Bank) was used as the primary antibody and goat anti-mouse rhodamine (red) was used as the secondary antibody. For the mineralization and protein adsorption tests, nuclei were stained with Hoechst stain (blue). The qualitative and quantitative measurements of cell viability on the ENF treated PMMA surfaces were conducted from images captured with an Olympus DP72 camera and CelSens software. Cell adhesion on the surface of all types of PMMA samples was analyzed for the qualitative measurement of cell viability. The number of cells adhered and the number of cells proliferated after adhesion to each sample were determined from the captured images using the ImageJ software program (http://imagej.nih.gov/ij/) (National Institutes of Health, Bethesda, MD, USA). Cell densities on ENF coated PMMA samples were compared with the control PMMA samples for the quantitative measurement of cell adhesion and the percentage of proliferation. The ratio of mineralized and osteonectin stain area over the total area of the image field was used to compare the mineralization and osteonectin activities between the control and ENF coated PMMA samples, respectively.

#### 4.3.3. Mechanical Tests

**Figure 13:**
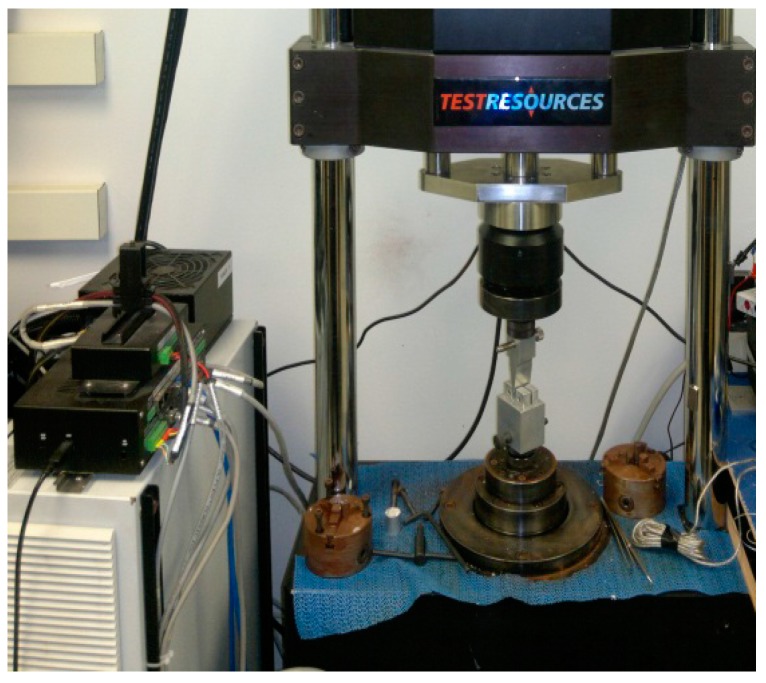
Three-point bend fixture for determining the bending properties of the bone cement.

Three-point bend (3PB) tests were conducted on each group of PMMA samples (*n* = 10) to compare the bending modulus, strength, and maximum deflection between the sample groups. Each sample was tested at room temperature at the loading rate of 0.01 mm/s using a Test Resources Universal Testing Machine. The specimens were mounted on the custom-made roller supports (span length = 16 mm) in the test stage and were pressed by a custom made 3PB indenter (tip radius = 3.2 mm) (Figure 13). The load vs. displacement was continuously recorded until the failure of the specimens. The corresponding stress-stress values from the load-displacement values and the bending properties were calculated using ASTM F417-78 standard formulations [29].

#### 4.3.4. Statistical Analysis

Independent sample *t*-tests, assuming unequal variances, were used to test for differences in mean adhesion density, cell proliferation, mineralization, amount of osteonectin, roughness, width, height, bending modulus, bending strength, and maximum deflection between the control and ENF coated PMMA samples. To test for differences in the mean number of adhered cells after 2 and 14 days, a two-factor (time and treatment) analysis of variance (ANOVA) was performed. Due to the presence of one extreme outlier and an indication of heterogeneity, ANOVA was performed on the log transformed data. A significance level of 0.05 was used for all tests. All analyses were performed using either proc *t* test or proc mixed in SAS v. 9.4 (SAS Institute, Cary, NC, USA).

## 5. Conclusions

This study found a statistically significant improvement on the osteoblast cytocompatibility properties of PMMA for PCL ENF treated PMMA samples compared to the non-treated PMMA samples due to the increased PMMA cement surface roughness. In addition, this study observed that the PCL ENF coating on PMMA had no adverse effect on the mechanical properties of PMMA under bending. Since the PCL ENF coating method developed in this study improved the physico- and biocompatibility of PMMA, it can be concluded that PMMA surface modifications by PCL ENF coating favor in vivo bone formation that leads to improved implant union with bone.

## Figures and Tables

**Table 1:** Difference of roughness between control and electrospun nanofiber (ENF) coated Poly(methyl methacrylate) (PMMA) samples. Data are presented as mean ± standard error. Data are presented with *n* = 3 for both samples. Note: *p* < 0.05 is denoted by * and *p* < 0.01 by ** (compared to control).

Descriptions	Control	ENF Coated	*t*	*p*-Value
*R_a_* (μm)	0.26 ± 0.03	2.75 ± 0.17 **	14.82	0.003
*R_z_* (μm)	1.45 ± 0.25	14.13 ± 1.24 **	10.02	0.008
*R_sum_* (μm)	75.47 ± 3.57	102.00 ± 6.30 *	3.66	0.032

**Table 2:** Summary statistics for the cell viability tests with respect to the cell culture time by sample group. Data are presented as mean ± standard error. Data are presented with *n* = 28 for both samples. Note: *p* < 0.05 is denoted by * and *p* < 0.01 by ** (compared to control).

Parameters Descriptions	Control	ENF Coated	*F*_1,56_	*p*-Value
No. of adhered cells after 2 days of cell culture	232 ± 16	309 ± 12 *	6.01	0.018
No. of adhered cells after 14 days of cell culture	428 ± 53	615 ± 65 **	10.48	0.002

**Table 3:** Summary statistics for the three-point bend test experimental data by sample group. Data are presented with *n* = 10 for both samples. Data are presented as mean ± standard error.

Parameters Descriptions	Control	ENF Coated	*t*	*p*-Value
Width of the sample	1.97 ± 0.01	1.98 ± 0.03	0.23	0.83
Height of the sample	1.49 ± 0.01	1.51 ± 0.01	1.31	0.20
Bending modulus (GPa)	3.32 ± 0.11	3.30 ± 0.07	0.15	0.88
Bending strength (MPa)	106.97 ± 4.94	108.12 ± 3.05	0.20	0.85
Maximum deflection (mm)	1.56 ± 0.07	1.58 ± 0.04	0.15	0.88
